# The Critical Studies of Fucoxanthin Research Trends from 1928 to June 2021: A Bibliometric Review

**DOI:** 10.3390/md19110606

**Published:** 2021-10-26

**Authors:** Yam Sim Khaw, Fatimah Md. Yusoff, Hui Teng Tan, Nur Amirah Izyan Noor Mazli, Muhammad Farhan Nazarudin, Noor Azmi Shaharuddin, Abdul Rahman Omar

**Affiliations:** 1 Laboratory of Aquatic Animal Health and Therapeutics, Institute of Bioscience, Universiti Putra Malaysia, Serdang 43400, Selangor, Malaysia; yskhaw@gmail.com (Y.S.K.); huiteng.tan28@gmail.com (H.T.T.); nuramirahizyan@gmail.com (N.A.I.N.M.); m_farhannaza@upm.edu.my (M.F.N.); 2 Department of Aquaculture, Faculty of Agriculture, Universiti Putra Malaysia, Serdang 43400, Selangor, Malaysia; 3 International Institute of Aquaculture and Aquatic Sciences, Universiti Putra Malaysia, Port Dickson 71050, Negeri Sembilan, Malaysia; 4 Department of Biochemistry, Faculty of Biotechnology and Biomolecular Sciences, Universiti Putra Malaysia, Serdang 43400, Selangor, Malaysia; noorazmi@upm.edu.my; 5 Laboratory of Vaccines and Immunotherapeutic, Institute of Bioscience, Universiti Putra Malaysia, Serdang 43400, Selangor, Malaysia; aro@upm.edu.my

**Keywords:** microalgae, macroalgae, carotenoids, fucoxanthin, Scopus, bibliometric, applications, biosynthesis, health benefits, production

## Abstract

Fucoxanthin is a major carotenoid in brown macroalgae and diatoms that possesses a broad spectrum of health benefits. This review evaluated the research trends of the fucoxanthin field from 1928 to June 2021 using the bibliometric method. The present findings unraveled that the fucoxanthin field has grown quickly in recent years with a total of 2080 publications. Japan was the most active country in producing fucoxanthin publications. Three Japan institutes were listed in the top ten productive institutions, with Hokkaido University being the most prominent institutional contributor in publishing fucoxanthin articles. The most relevant subject area on fucoxanthin was the agricultural and biological sciences category, while most fucoxanthin articles were published in *Marine Drugs*. A total of four research concepts emerged based on the bibliometric keywords analysis: “bioactivities”, “photosynthesis”, “optimization of process’’, and “environment”. The “bioactivities” of fucoxanthin was identified as the priority in future research. The current analysis highlighted the importance of collaboration and suggested that global collaboration could be the key to valorizing and efficiently boosting the consumer acceptability of fucoxanthin. The present bibliometric analysis offers valuable insights into the research trends of fucoxanthin to construct a better future development of this treasurable carotenoid.

## 1. Introduction

Fucoxanthin is an orange-colored pigment, which accounted for more than 10% of the estimated total carotenoids production in the nature [1]. It is the most abundant and signature carotenoid in brown macroalgae and diatoms [2]. It is a part of fucoxanthin–chlorophyll *a* protein (FCP) complexes along with proteins and chlorophyll *a* (chl *a*) that are functionally associated with the light-harvesting complex of the algae [3]. 

It has been shown that fucoxanthin possesses numerous biological activities and health-stimulating properties such as anti-angiogenic, anti-diabetic, antioxidant, anti-inflammatory, and antimalarial activity [4,5]. Notably, in vivo studies (mice and human) have proven that fucoxanthin effectively decreases body weight [6,7]. In addition, fucoxanthin has been demonstrated to exhibit a wide range of anticancer and anti-proliferative activity in different in vivo and in vitro studies [8]. These bioactivities of fucoxanthin are attributed to its unique molecular structure such as an allenic bond, an acetyl group, and a conjugated carbonyl with a 5,6-monoepoxide [9]. Due to its multiple health benefits, the global total fucoxanthin market is estimated to increase from $88 million in 2019 to more than $100 million over the next five years (Global Fucoxanthin Market 2020). 

Bibliometric analysis is an approach that analyzes the worldwide scientific production and qualitative data. The analysis could provide information on the research trends and future directions within a specific research field [10]. Lately, a bibliometric analysis of microalgae research, microalgae-based wastewater treatment, global dinoflagellate research, and microalgae-derived pigments has been performed [10,11,12,13]. These bibliometric analyses focused on microalgae or metabolites generated from microalgae. Silva et al. (2020) [12] only reviewed four pigments, chlorophylls, phycocyanin, and β-carotene of the microalgae using a bibliometric approach. A critical examination of the research trends of fucoxanthin utilizing the bibliometric method is still lacking. Thus, this review aimed to analyze the research trends of the fucoxanthin field via bibliometric analysis. Several conclusions can be drawn through the analysis, such as evaluating essential milestones over the history of a scientific field and the scientific fads. The keyword analysis in the current review highlighted the trends and directions of fucoxanthin research. The review could serve as a basis in fortifying the future direction of fucoxanthin research. 

## 2. Methods

Bibliometric analysis in the present study employed three-step approaches. These steps were data mining, data refinement, and data analysis.

### 2.1. Data Mining

A set of publications associated with fucoxanthin research was first extracted from the Elsevier Scopus database (collected on 15 July 2021). A complete search of the database was performed using [TITLE-ABS-KEY (fucoxanthin)] as the search query. This query retrieved a total of 2080 publications. Different search results will be obtained if distinct search parameters or databases were utilized. 

### 2.2. Data Refinement

Data refinement is indispensable in bibliometric study, as it could remove the irrelevant articles. The retrieved data were polished using OpenRefine software, which excluded duplicate articles [11]. In addition, each article was also examined meticulously by manual inspection to eliminate unrelated articles. 

### 2.3. Data Analysis

The information of publications was manually extracted. For example, journal impact factors (IFs) were retrieved from Journal Citation Reports (JRC) in 2020. In addition, the number of total citations and the h-index value were obtained from the Scopus database (https://www.scopus.com/search/form.uri?display=basic#basic; collected on 15 July 2021). On the other hand, software such as VOSviewer was utilized to perform bibliometric analyses. VOSviewer was employed to visualize the similarities among different subjects (countries and keywords) [14]. In addition, the visualization map was generated using VOSviewer to display the relationship between subjects in a research field. The research network and keyword co-occurrence network were constructed and visualized with VOSviewer. In the case of the keyword co-occurrence network, the most commonly utilized keywords related to the searched words (fucoxanthin) were screened. The minimum number of occurrences for the keyword co-occurrence network was determined as 15. Manual inspection was performed to eliminate the unrelated keywords. Moreover, the data were analyzed using the bibliometric R-package incorporated in the open-source RStudio software (www.rstudio.com; version 4.0.5, assessed on 15 July 2021) [15]. 

## 3. Publication Output

**Figure 1:**
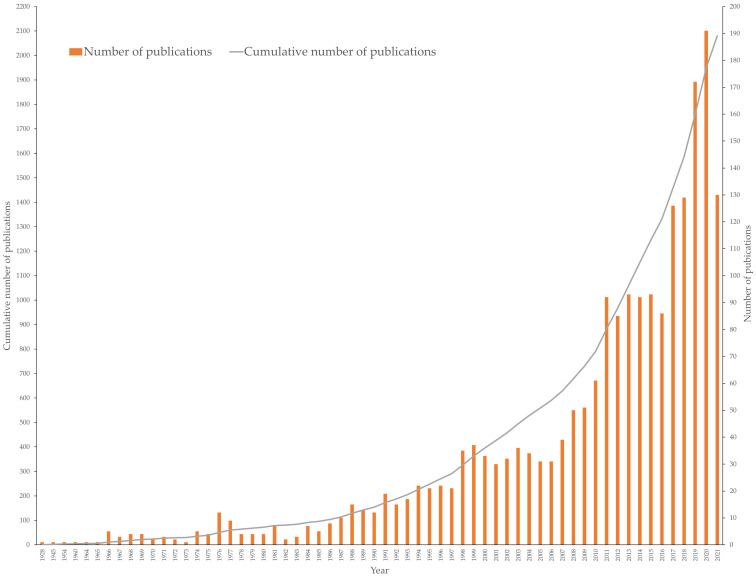
The annual and cumulative numbers of fucoxanthin publications from 1928 to June 2021.

A total of 2080 fucoxanthin research articles were published from 1928 to June 2021 (Figure 1). According to the Scopus database, the first fucoxanthin research article was published in 1928. A small number of publications per year were observed (<10 units except 1976) until 1986. The amount of publication has doubled (around 20 units) per year since 1994. After that, the annual publications have elevated steadily and generated an immediate increase in the cumulative total publications. A remarkable increase in the number of publications was detected starting in 2017. In 2017, the number of publications exceeded 100 for the first time. The last decade’s publication on fucoxanthin corresponded to 64.90% (1350 publications) of the total publications since 1928. In addition, the previous years (2017 to June 2021) recorded 35.96% of the total publications on fucoxanthin. These findings indicated that a strong interest in fucoxanthin research occurred recently. 

**Figure 2:**
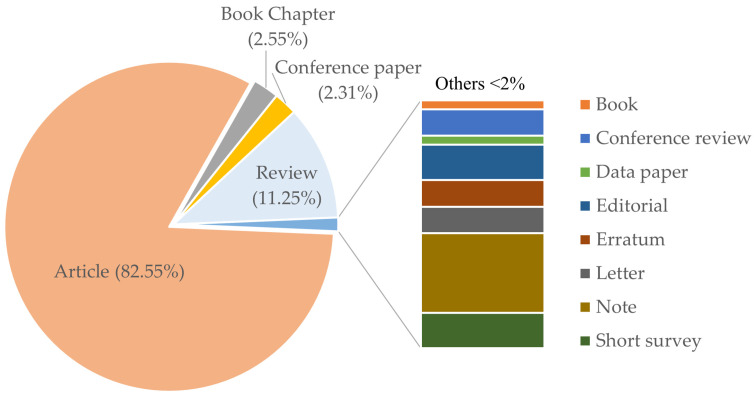
Distribution of publication types for fucoxanthin.

The major publications on fucoxanthin were articles (82.55%) (Figure 2). Reviews were the second-largest amount of publications, which constituted 11.25% of the total publications. This is followed by book chapters (2.55%) and conference papers (2.31%). Other documents such as books, conference reviews, data papers, editorials, errata, letters, notes, and short surveys accounted for less than 2% of the total publications.

## 4. Global Production of Fucoxanthin Publications

Fucoxanthin studies have been conducted over the continentals in the world. The top ten countries have contributed to more than 70 publications from 1928 to June 2021 (Table 1), indicating these countries are the key players in studying fucoxanthin. From the time perspective, the United States predominates in producing fucoxanthin articles among the other countries from 1928 to 2000. Although more articles (*n* = 115) by the United States from 2001 to June 2021 were observed compared to the previous period, the United States could only be classified as a second or third place among the other countries in the last two periods. All these top ten countries demonstrated noticeably increased research performance on fucoxanthin from 1928 to June 2021. The most apparent upsurge of fucoxanthin publications was seen in 2011–June 2021 for the top ten countries except for Canada. For example, China showed the most remarkable productivity (almost 24-fold) on fucoxanthin articles; the number of articles escalated from nine (2001–2005) to 219 (2011–June 2021). Overall, Asian countries focused on fucoxanthin publications more than the other countries, as four Asian countries (Japan, China, South Korea, and India) occupied the top ten countries (1928–June 2021). The major three countries that produced the most fucoxanthin publications were Japan, the United States, and China. Japan leads the first ranking in the publications on fucoxanthin with 379 articles, suggesting that Japan plays a significant role in advancing fucoxanthin research. 

The top ten institutions contributed at least 33 fucoxanthin publications, respectively (Table 2). These top institutions were leaded by Hokkaido University, Japan (110 articles), CNRS Centre National de la Recherche Scientifique, France (61 articles), and Goethe-Universität Frankfurt am Main, Germany (49 articles). Among these ten institutions, five were European and five were Asian. Institutions from Japan occupied three of the top ten institutions: Hokkaido, Kyoto, and Kobe University. The contribution of these three major Japanese institutions resulted in Japan predominating in fucoxanthin research. Fewer fucoxanthin publications were produced from these top ten institutions in 1928–1990 except for Norges teknisk-naturvitenskapelige universitet, Norway. Most of the top ten institutions increased their publications at least one-fold from the 2001–2010 period to the 2011–June 2021 period. The most remarkable elevation of fucoxanthin publications was produced by the Chinese Academy of Sciences, China (around 18.5-fold). 

**Figure 3:**
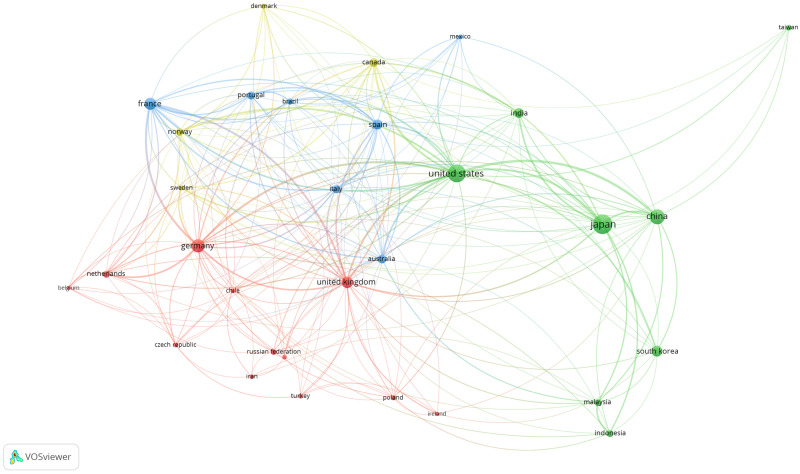
Visualization map of countries communities in fucoxathin research (minimum occurrence: 20).

A distribution by communities of 31 countries with at least 20 articles on fucoxanthin was observed (Figure 3). These countries were distributed over four clusters. The first cluster comprised of eight European countries, along with Chile, Iran, Turkey, and New Zealand. The second cluster consisted of Mexico, Australia, Brazil, and four European countries. The third cluster was made of the United States and seven Asian countries, while Canada and three European countries constituted the last cluster. Based on Figure 3, the United Kingdom established a research collaboration with the highest number of countries (26 countries). The United States was the second country that formed the most partnerships with other countries (25 countries). Germany had the third highest number of country collaborations (23 countries). For the strength of research collaboration, the United States formed the most substantial collaboration network with other countries (total link strength: 214). This is followed by Germany (126), the United Kingdom (125), France (118), and Japan (103). 

The top ten productive authors produced at least 18 publications (Table 3). Eight of the top ten productive authors were affiliated with Japanese institutions. No publication was generated from these top ten authors in the first period (1928–1970). The number of the publication produced by Liaaen-Jensen, Synnove (Germany) outweighed the other authors from the second period (1971–1980) to the fourth period (1991–2000). The last two periods were dominated by Miyashita, Kazuo (Japan) in terms of publications. Miyashita Kazuo was the most productive and impactful author on fucoxanthin research with 88 publications and a Scopus *h*-index of 50. The *h*-index was utilized to evaluate a researcher’s contribution. The *h*-index not only indicates productivity, it also demonstrates the influence of a group’s or scholar’s published work [16]. The author recently studied the effect of fucoxanthin/fucoxanthinol on cancer, particularly pancreatic and colon cancer [17,18]. Masashi Hosokawa produced 68 publications and became the second most productive author on fucoxanthin research (Scopus *h*-index: 44). The author worked together with Kazuo Miyashita in examining the effect of fucoxanthin on dexamethasone-induced muscle atrophy and fat mass in mice [19]. The third most productive author was Claudia Buchel (41 publications, Scopus *h*-index: 34), who has investigated the structure and energy transfer of FCP complexes lately [20,21]. Synnove Liaaen-Jensen was among the leading researchers from Norway. The author demonstrated the iodine-catalyzed R/S isomerization of the allenic carotenoids fucoxanthin via the attack of I·on C7′ [22]. In addition, the author also studied the diphenyl diselenide-mediated photoisomerization of allenic carotenoids fucoxanthin in benzene solution to optimize synthetic yields of (6′S)-allenes [23]. A team led by Ryo Nagao studied the structure and function of a diatom photosystem I-light-harvesting supercomplex [24] and adaptation of light-harvesting and energy-transfer processes of diatoms [25,26]. 

Multidisciplinary collaboration networks among researchers are crucial in developing, evolving, and enriching the nature of fucoxanthin utilization research. The high productivity of these top ten authors could be attributed to the formation of effective research teams via co-authorship networks. The homophyllous network is a collaboration among researchers of the same gender, academic department, and research interest. This network is vital in establishing and sustaining the research capabilities of the members of the organization. On the other hand, heterophyllous collaboration allowed the cooperation among researchers of different gender, affiliations, and research interests. This cooperation could provide new insights or solutions to complex problems and create transformative research [27]. 

## 5. Subject Category, Sources and Citations

At least 2.0% of total publications were assigned to each of the top ten subject categories for fucoxanthin research (Table 4). The global fucoxanthin publication landscape covered 27 subject categories. A total of 27.0% publications was classified under the agricultural and biological sciences category. This category’s highest percentage of publications signifies the solid scientific interests in the fundamental knowledge of agricultural utilization [28], synthesis, and accumulation [29,30] of fucoxanthin. The rapid development of biochemistry [24,31] and molecular techniques such as genetic transformation [32,33], sequencing [34], and transcription [35] led to the dominance of biochemistry, genetics, and molecular biology category (18.1% publications). The high number of publications in the pharmacology, toxicology, and pharmaceutics (8.3% publications) category could be due to the increase of health-consciousness and the benefits of fucoxanthin [36,37]. However, a low percentage of articles in the economic and social science categories (data not shown) implies that fucoxanthin research is still in its infancy. Thus, it is crucial to focus on the economic viability and social acceptance of fucoxanthin in the future. 

A total of 689 scientific journals published fucoxanthin articles from 1928 to June 2021. The top ten journals on fucoxanthin research belonged to seven different publishers (Table 5). Springer occupied three of the top ten journals. The rest of the journals were owned by Multidisciplinary Digital Publishing Institute (MDPI), Wiley Periodical LLC, Elsevier B.V., Inter-Research Science, American Chemical Society, and Oxford University Press. According to the InCites Journal Citation Reports, the top ten journals showed at least an impact factor of 2.400. The journal with the highest impact factor belonged to the Journal of Agricultural and Food Chemistry (5.279), while the Journal of Plankton Research was the lowest impact factor journal (2.455) among the top ten journals. 

Articles from these ten top journals constituted 22.20% of the total fucoxanthin publications (Table 5). The most productive journal was *Marine Drugs*, with 123 articles covering 5.91% of the total publications. This is followed by the *Journal of Phycology* (67 articles, 3.22%), *Journal of Applied Phycology* (55 articles, 2.64%), *Photosynthesis Research* (39 articles, 1.88%) and *Biochimica Et Biophysica* *Acta* (BBA)—*Bioenergetics* (38 articles, 1.83%). 

The top ten articles in Table 6 demonstrated at least 178 citations. The data of highly cited publications are impactful, as these mirror the scientific advancement, offer revolutionary insights, and provide a significant perspective on scientific advancement [38]. Each of these articles was owned by one prestigious journal except *Marine Drugs* and the *Journal of Agricultural and Food Chemistry*. *Marine Drugs* possessed two top fucoxanthin publications with a high total citation: Peng et al. (2011) and Xia et al. (2013), while two highly cited fucoxanthin research articles were published in *Journal of Agricultural and Food Chemistry*: Sachindra et al. (2007) and Maeda et al. (2007). Furthermore, the top ten most cited publications were observed in only two of the top ten journals, namely *Marine Drugs* and *Journal of Agricultural and Food Chemistry*. The research article produced by Maeda et al. (2005) in *Biochemical and Biophysical Research Communications* was the publication that received the highest total citation on fucoxanthin research (411). The article demonstrated that fucoxanthin upregulates the expression of mitochondrial uncoupling protein 1 that may attribute to the reduction of white adipose tissue (WAT) weight [39]. The review article summarizes the topical state of understanding and provides an overview of the basic knowledge of fucoxanthin. The second highly cited publication was a review article (356). This review discussed the metabolism, safety, and bioactivities of fucoxanthin [2]. The other research article discovered that fucoxanthin was the major active compound in Japanese edible macroalgae, *Hijikia fusiformis*, which might be responsible for the high antioxidant activity [40]. This article was ranked third in the top ten most-cited publications (348). Most of the top ten cited fucoxanthin articles described and discussed the bioactivities of fucoxanthin (i.e., antiobesity, antioxidant, antiproliferation, anti-inflammatory) [2,6,39,40,41,42,43,44]. This could be inferred as the interest of researchers was focused on the bioactivities of fucoxanthin lately.

## 6. Research Concepts

**Figure 4:**
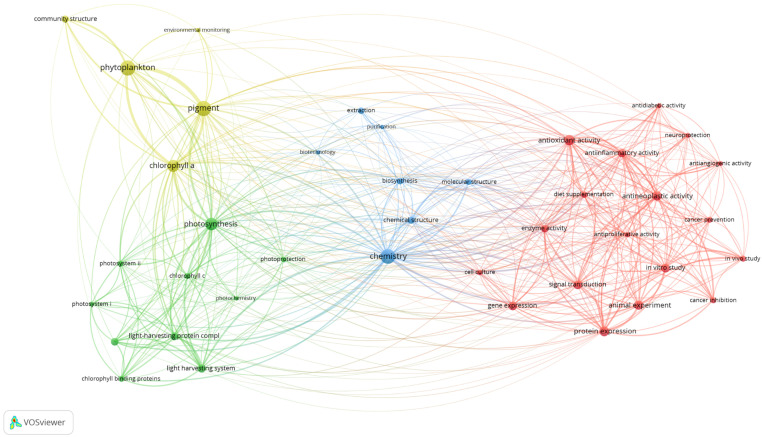
Visualization of keyword co-occurrence network analysis (minimum occurrences: 15).

The keyword co-occurrence analysis provides an overview of fucoxanthin research concepts. Keywords serve as the fundamental of bibliographic research of academic literature [45]. A total of 40 relevant keywords were classified into four different clusters using a minimum occurrence of the 15-fold keyword (Figure 4). The first cluster (in red) consists of 18 keywords that revolved around “antioxidant activity”, “neuroprotection”, and “cancer inhibition”. This cluster highlighted the bioactivities of fucoxanthin via in vitro study and in vivo study. Ten keywords such as “photosynthesis”, “photosystem”, “chlorophyll-binding proteins”, and “energy transfer” were seen in the second cluster (in green). This cluster represents the research of photosynthesis that involved fucoxanthin. The third cluster (in blue) encompassed “chemistry”, “biosynthesis”, “extraction”, “purification”, and “biotechnology” keywords. Thus, this cluster depicted the optimization of processes in obtaining fucoxanthin as valuable compounds. The fourth cluster (in yellow) revolved around the major keywords such as “phytoplankton”, “community structure”, “pigment”, and “environmental monitoring”, which denoted the role of fucoxanthin in the environmental study of phytoplankton. 

### 6.1. Research Trends of Fucoxanthin Research from 1928 to June 2021

**Figure 5:**
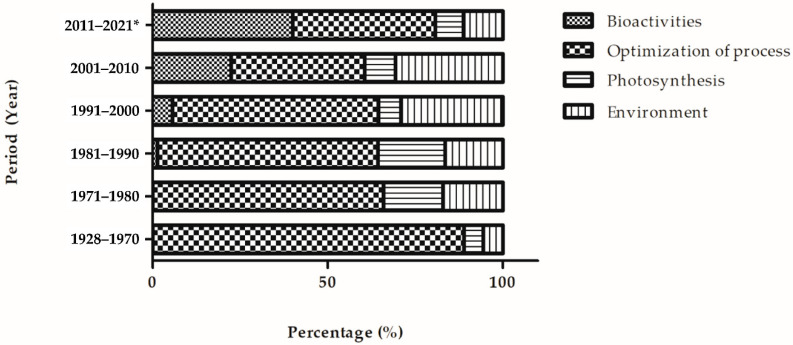
Research trends of fucoxanthin research from 1928 to June 2021. The asterisk after 2021 denotes that the data were extracted up to June 2021.

The four clusters (bioactivities, photosynthesis, optimization of process, environment) were utilized to analyze the fucoxanthin research trends from 1928 to June 2021. In the first period (1928–1970), the researchers focused on the optimization of process cluster (88.88%) (Figure 5). An equal percentage of fucoxanthin articles (5.56%) was observed in photosynthesis and environment clusters, respectively. No bioactivities of fucoxanthin publication were produced. In the second period (1971–1980), the percentage of articles in the optimization of process cluster decreased by 22.92% compared to the previous period. Both photosynthesis and environment clusters demonstrated the same percentage of publications, 17.02% (increased by 11.46%). The bioactivities cluster remained 0%. There was a further reduction (2.95%) of publications in the optimization of process cluster in the third period (1981–1990) compared to the previous period. In the same period, the bioactivities, photosynthesis, and environment clusters increased the percentage of publications, 1.37, 2.16, and 1.42%, respectively.

In the fourth period (1991–2000), bioactivities and environment clusters experienced an increase of publications percentage, 4.26, and 12.66%, respectively, compared to the previous period. On the other hand, the optimization of process and photosynthesis clusters exhibited a fall of publications percentage, 4.13, and 12.69%, respectively. In the fifth period (2001–2010), the only optimization of production cluster experienced a reduction of articles percentage to 38.13% compared to the earlier period. The remaining clusters displayed an increase in articles percentage. The biggest upsurge in terms of publications percentage (16.77%) was detected in the bioactivities cluster among the other clusters. The photosynthesis cluster showed 8.8% of publications, while 30.67% of publications were identified in the environment cluster. In the sixth period (2011–June 2021), the bioactivities cluster (39.97%) demonstrated a similar publications percentage with the optimization of process cluster (40.79%). The photosynthesis cluster displayed 8.03% of publications, whereas 11.22% of publications were seen in the environment cluster. Throughout the six periods (1928–June 2021), the interest in fucoxanthin research shifted from the optimization of the processing cluster to the bioactivities cluster. This could be because brown macroalgae and microalgae are considered a potential natural source of bioactive compounds (i.e., fucoxanthin), and these compounds provide various health benefits [46]. 

#### 6.1.1. Bioactivities of Fucoxanthin 

The interest in natural sources of bioactive compounds is growing immensely due to the numerous benefits to humans. According to the Scopus database, the first article on the bioactivities of fucoxanthin was published in 1990. In the first article on the bioactivities of fucoxanthin, Okuzumi et al. (1990) demonstrated the anti-tumor activity of fucoxanthin (isolated from brown algae, *Hijikia fusiforme*) on human neuroblastoma cells, GOTO cells. The authors showed that fucoxanthin caused the arrest in the G_0_–G_1_ phase of the cell cycle. In addition, fucoxanthin decreased the expression of the N-myc gene, and the authors suggested this mechanism could prevent the proliferation of cancer cells [47]. In the next several years, Okuzumi et al. (1993) showed that fucoxanthin inhibited duodenal carcinogenesis induced by N-ethyl-N’-nitro-N-nitrosoguanidine in mice [48]. Fucoxanthin was documented as a chemopreventive agent in the first bioactivities of fucoxanthin review based on Scopus search results [49]. 

The bioactivities of fucoxanthin have been extensively studied. The bioactivities of fucoxanthin in humans have been summarized in many review articles [2,4,5,8,50,51,52,53]. For instance, Peng et al. (2011) [2] reviewed fucoxanthin’s metabolism, safety, and bioactivities. The bioactivities include antioxidant, anti-inflammatory, anti-obese, anti-diabetic, antimalarial, anticancer, and anti-angiogenic activity and hepatoprotective, skin protective, cerebrovascular protective, bone protective, and ocular protective effects [2]. Furthermore, Thiyagarasaiyar et al. (2020) [5] discussed the cosmeceutical potentials of fucoxanthin as skin whitening, anti-aging, anticancer, antioxidant, anti-inflammation, and antimicrobial. In addition, Sathasivam and Ki (2018) [54] summarized the publications on anti-angiogenic, anticancer, antidiabetic, anti-obesity, and antioxidant activity and then on the neuroprotective, cardioprotective, and osteo-protective effect of fucoxanthin. Despite these bioactivities, the mechanism of these bioactivities also has been reviewed. For example, Rengarajan et al. (2013) [55] summarized the mechanisms of anticancer effects of fucoxanthin. These mechanisms were anti-proliferation, induction of apoptosis, cell cycle arrest, and anti-angiogenesis. Moreover, the mechanisms of action of fucoxanthin on different types of cancers were also elucidated [56]. Additionally, the modulation of inflammatory and oxidative stress pathways using fucoxanthin was described [57]. Another review article summarized the possible underlying mechanism of fucoxanthin on lipid metabolism, adiposity, and related conditions [58]. 

The combined effect of fucoxanthin and the other compound(s) were also studied. Maeda et al. (2007) [6] demonstrated that feeding both fucoxanthin and fish oil to KK-*A^y^* mice significantly reduced the WAT weight of mice compared with the mice fed fucoxanthin alone. Moreover, the reduction of serum levels of triacylglycerols, glucose, and leptin in diet-induced obese rats was observed using a combination of fucoxanthin and conjugated linoleic acid [59]. A combination of fucoxanthin and vitamin C has been shown to increase human lymphocytes’ antioxidant and anti-inflammatory effects [60]. Another study demonstrated the combined effects of low-molecular-weight fucoidan and fucoxanthin in a mouse model of type II diabetes. The authors reported that the combination effectively decreased the urinary sugar, glucose, and lipid metabolism in the WAT of the mice than fucoidan or fucoxanthin alone [61]. The effects of fucoidan and fucoxanthin in combination were investigated in aging mice and hyperuricemic rats. The combination of these compounds improved the cardiac status of aging mice via decreased cardiac hypertrophy, cardiac fibrosis, reactive oxygen species level, and shortened QT interval in the mice [62]. For hyperuricemic rats, the combination inhibited xanthine oxidase activity in the liver and controlled the expression of uric acid-related transporters [63]. Furthermore, a combination of fucoxanthin and rosmarinic acid could offer photo-protective effects through the downregulation of NRLP3-inflammasome and increasing the Nrf2 signaling pathway in UVB-irradiated HaCaT keratinocytes [64]. Recently, a combination of fucoxanthin, myoinositol, D-chiro-inositol, and hydroxytyrosol has been reported to decrease systolic blood pressure and improve the vascular reactivity in a pregnant mouse model of hypertension [65]. 

Apart from this, the bioactivities of fucoxanthin were also studied in the other organisms. Fucoxanthin enhanced the phagocytic activities three times and increased the number of ovulated eggs of sea urchins by 3.25 times compared to the control group [66]. The planktonic larvae stage is crucial in the life cycle of coral. The percentage of a larval metamorphosis of coral *Pseudosiderastrea tayamai* was further enhanced by 60.3% in the presence of fucoxanthin compared to control group [67]. The effect of fucoxanthin was also examined in fly and worm. The median and maximum lifespan of *Drosophila melanogaster* (fly) was extended at least 33% and 12%, respectively using fucoxanthin. The decreased flies’ fecundity, increased spontaneous locomotor activity, and resistance to oxidative stress were observed when feeding the flies with fucoxanthin. For *Caenorhabditis elegans* (worm), the feeding of fucoxanthin increased the mean and maximum lifespan by 14% and 24%, respectively [68]. 

#### 6.1.2. Photosynthesis 

Diatoms and brown algae utilized unique light-harvesting antennas, FCPs, to perform photosynthesis [69]. The pigment compositions and protein organization of FCPs are mostly distinct from those of light-harvesting complexes (Lhcs) in land plants and green algae [70,71]. At least 30 genes related to light harvesting have been identified based on the genome of two diatoms, *Thalassiosira pseudonana* [72] and *Phaeodactylum tricornutum* [73]. The products encoded by these genes were categorized into three groups, Lhcf, Lhcr, and Lhcx proteins. Proteins under the Lhcf family are the main light-harvesting proteins [74]. PSI antennae are composed of proteins from the Lhcr family [3], while Lhcx proteins are involved in non-photochemical quenching mechanisms to protect the photosystem [75]. A high similarity of Lhcx proteins to LI818 or LhcSR proteins of green alga was observed [76]. 

The FCP trimer, also known as FCPa, is the basic structure of different FCP proteins in the native thylakoid membrane of pennate and diatoms [77]. Diverse populations of FCP trimeric complexes, which differed in polypeptide composition and pigmentation, were identified through the sub-fractionation of FCP complexes of *P. tricornutum* [78]. Four different trimeric subtypes of FCPa in *Cyclotella meneghiniana* were revealed, FCPa1–4. Lhcf4/Lhcf6 proteins were found mainly in the FCPa2 trimer, whereas Lhcf1 was reported to be the major subunit in FCPa1, FCPa3, and FCPa4 [79]. Buchel (2003) [80] discovered that two FCP fractions differed in the polypeptide composition and oligomeric state from *C. meneghiniana*. The first fraction consisted of trimers with mainly 18 kDa polypeptides (FCPa), while the higher oligomers assembled from different trimers (19 kDa subunits) constituted the second fraction (FCPb). Two oligomeric subtypes, FCPb1 and FCPb2, with Lhcf3 being the main subunit in both antenna complexes in *C. meneghiniana* were revealed [79]. Different FCP complexes were observed using anion chromatography and divided into FCP complexes related to PSI, PSII core complexes, and peripheral FCP complexes. Various Lhcf proteins were detected in FCP complexes associated with PSI and PSII core complexes, whereas peripheral FCP complexes mainly contained Lhcf8 and Lhcf9. Subunits of the PSI core complex composed of Lhcr proteins and Lhcx proteins were the protein subunits that were identified in the PSII core complex [81]. 

The idea of the FCP trimer as the basic unit of photosynthesis antenna proteins in fucoxanthin-containing algae was contradicted by the findings based on cryo-electron miscopy [82,83,84,85] and X-ray crystallography [86]. Wang et al. (2019) [86] unraveled the X-ray crystal structure of an FCP of *P. tricornutum,* which had two monomers held together to form the dimeric structure of FCP within the PSII core. In addition, the cryo-electron microscopy data of the PSII–antenna supercomplex of *Chaetoceros gracilis* revealed a tetrameric organization of FCP proteins associated with the PSII [82]. Furthermore, 24 FCPs surrounding the PSI core of *C. gracilis* were in monomeric form based on cryo-electron microscopy [83]. 

Important characteristics of pigment organization of isolated FCP and the role of fucoxanthin molecules in excitation energy transfer have been unraveled using steady-state and ultrafast spectroscopic methods [87,88]. Efficient energy transfer was observed from fucoxanthin and chlorophyll *c* (Chl *c*) to Chl *a* based on spectroscopic studies [87,89,90]. At least three forms of fucoxanthin molecules differ in their photophysical and dipolar properties, Fx_red_, Fx_green_, and Fx_blue_ [91,92] and were confirmed using resonance Raman spectroscopy [93]. The Fx_red_ form transfers energy more efficiently, while the Fx_blue_ form demonstrated less efficiency in transferring excitation energy [92]. The time of energy transfer from fucoxanthin to Chl (around 300 fs) was shorter than the transfer from Chl *c* to Chl *a* (around 500 fs–6 ps), indicating that the fastest energy transfer was between fucoxanthin and Chl *a* [94]. Most of the pump-probe studies examined the dynamical energy transfer process in FCP of fucoxanthin-containing algae. For example, Papagianakis (2005) [87] characterized the energy transfer network in FCP. The energy transfer efficiency from Chl *c* to Chl *a* is 100%, whereas unequal efficiency was observed for fucoxanthins in the FCP. Furthermore, findings based on polarized transient absorption indicated that three fucoxanthin molecules in FCPa transferred their excitation energy directly to Chl *a*. The remaining fucoxanthin molecule was not associated with Chl *a* molecules and might transfer its excitation energy through another fucoxanthin molecule to Chl *a* [89]. In addition, a detailed model was proposed in describing the energy transfer in FCPa upon excitation at two different wavelengths [95]. Another study demonstrated the highly efficient energy transfer from Fx to Chl-a through the S_1_/ICT state using the pump-probe method [96]. Apart from pump-probe techniques, two-dimensional electronic spectroscopy (2DES) has offered insights into energy change transfer dynamics, exciton diffusion, and molecular system relations [97,98,99]. The advances in knowledge of the mechanisms and dynamics of energy transfer in the FCPs of diatoms that have been accomplished using two-dimensional electronic spectroscopy (2DES) were reviewed [100].

In addition to FCPs’ function as a light-harvesting complex, pigments in the FCPs are also involved in photoprotection. Non-photochemical quenching (NPQ) is the protection mechanism that most algal groups utilize to dissipate excess absorption energy as heat via molecular vibrations. One xanthophyll pigment, diadinoxanthin (Dd), was observed in a peripheral location of FCP [86]. This pigment is converted to diatoxanthin (Dt) under high light intensities [101]. The conversion showed a close relationship with the build-up of the NPQ mechanism [102]. Previously, the amount of diatoxanthin (Dt) was established to influence the NPQ mechanism in vivo, whereas the reduction of fluorescence yield of FCPa complexes in vitro was caused by Dt [103]. In addition, the acidification of thylakoid lumen regulated the ratio between Dd and Dt, which could affect the activities of the epoxidase and de-epoxidase in the NPQ mechanism [104]. Gundermann and Claudia (2012) [105] examined the factors determining the NPQ process in diatoms. The components involved in the NPQ mechanism in C. *meneghiniana* were reported to be heterogeneous and genuinely different from the NPQ type in *P. tricornutum* [106]. Apart from xanthophyll pigments (Dd and Dt), Lhcx proteins play an essential role in the NPQ mechanism [76,107]. The amount of xanthophyll cycle pigments in various FCP preparations showed a relationship with the existence of Lhcx1 protein [108]. This protein was shown to have short-term photoprotection [107], which induced the conformational changes of the FCPs and reduced fluorescence yield [109]. The molecular structure, the arrangement of the different Lhc proteins in the complexes, the energy transfer abilities, and the photoprotection of other Lhc systems of Chl *c* containing organisms were reviewed recently [110]. 

#### 6.1.3. Optimization of Process

The optimization of the process cluster focused mainly on the biosynthesis, biotechnology, extraction, and purification of fucoxanthin. Algal cultivation and fucoxanthin production are the major components of the biosynthesis section. Previous studies demonstrated that the fucoxanthin content of microalgae is higher than that of brown macroalgae [111,112]. The fucoxanthin extracted from fresh brown macroalgae ranged from 0.02 to 0.87 mg/g fresh weight, while the dried form of microalgae showed 1.81–15.33 mg/g dried weight (DW) fucoxanthin [111]. In diatoms, the fucoxanthin content ranged from 0.224% to 2.167% (around 0.224–21.67 mg/g) of dry weight [111,113]. Currently, *P. tricornutum* is the major natural fucoxanthin source in microalgae due to its substantial fucoxanthin [111,114]. Nevertheless, the lower dry well weight (g/L) of this microalgae [114] prompted the searching for alternative fucoxanthin sources via screening. The screening of high-performance microalgae strain is crucial, as it could determine the success of producing the desired amount of fucoxanthin prior to algal cultivation. Previous studies attempted to screen for potential microalgae with a high production of fucoxanthin [115,116]. For instance, Guo et al. (2016) [115] screened 13 diatoms strains to identify a promising strain with desired fucoxanthin production. Among these 13 diatoms strains, the highest fucoxanthin content was *Odontella aurita* (1.50% or 15.00 mg/g DW). A maximum biomass and fucoxanthin concentration of 6.36 g/L and 18.47 mg/g DW were reported for *O. aurita*, respectively [117]. Another study screened *Isochrysis* strains for their potential for concurrent DHA and fucoxanthin production [116]. *Isochyrsis* CCMP1324 demonstrated the comparable biomass concentration (2.72 g/L), DHA content (16.10 mg/g) and fucoxanthin content (14.50 mg/g). The accurate identification of microalgae is essential in the screening process to ensure repeatability, reproducibility, and quality assurance. A comprehensive identification method could assign a precise identity to the microalgae [118]. Successful algal cultivation is affected by the culture parameters. The past studies optimized several culture parameters to obtain the maximum amount of fucoxanthin [114,116]. For instance, McClure et al. (2018) [114] optimized the culture parameters such as light intensity, medium composition, and carbon dioxide addition on the fucoxanthin production of *P. tricornutum*. The authors obtained the maximum concentration of fucoxanthin (59.20 mg/g), which is nearly four times higher than that found by Kim et al. (2012) [111]. These parameters are required to optimize in order to develop a sustainable, feasible, and economically viable cultivation modus operandi for the microalgae [119]. 

Genetic transformation is one of the critical components of genetic engineering. Several genetic transformation protocols have been established for fucoxanthin-containing microalgae [120,121,122,123]. In addition, successful genetic engineering relies on a suitable promoter. Several past researchers searched for the promising promoter in enhancing the gene expression of fucoxanthin-containing algae [122,124]. For example, four different promoters were examined for the genetic transformation of brown algae [122]. The authors concluded that the FCP promoter of *P. tricornutum* was the most suitable promoter for the brown algae, as this promoter induced both integrated and transient expression in the algae. Erdene-Ochir et al. (2016) [124] discovered a potential endogenous promoter of *P. tricornutum*, which is a glutamine synthetase promoter. This promoter induced the gene expression constitutively, and it was at least four times higher than the FCP promoter at the stationary phase.

Furthermore, four additional novel promoters were found in *P. tricornutum* under varied nitrate availability [125]. Moreover, five putative endogenous gene promoters highly expressed in *P. tricornutum* were isolated [126]. The activity of the Vacuolar ATPase (V-ATPase) gene promoter was higher than the other promoters and could drive the gene expression under both light and dark conditions at the stationary phase. The overview of the fucoxanthin synthesis pathway is crucial in genetic engineering. Several review articles have discussed the biosynthetic pathway of fucoxanthin [127,128,129]. Previous studies adopted the genetic engineering of the carotenoid gene(s) to improve the fucoxanthin content [130,131]. For instance, the introduction of an additional endogenous 1-deoxy-D-xylulose 5-phosphate synthase and phytoene synthase (Psy) gene separately into the genome of *P. tricornutum* resulted in an elevation of carotenoids amounts such as fucoxanthin [130]. In addition, the overexpressing of the phytoene synthase gene (isolated from *P. tricornutum*) in *P*. *tricornutum* also enhanced the fucoxanthin content by around 1.45-fold compared to the wild-type diatom [131]. Recently, Manfellotto et al. (2020) [33] produced *P. tricornutum* transformants with the overexpression of violaxanthin de-epoxidase, Vde-related, and zeaxanthin epoxidase 3. These transformants demonstrated an increased accumulation of fucoxanthin content up to four-fold compared to the wild type.

The production of high-quality fucoxanthin depends on an effective extraction and purification method. Various protocols have been utilized to recover and purify fucoxanthin from algae. These included centrifugal partition chromatography [132], column chromatography [133], microwave irradiation [113], pressurized liquid extraction [134], supercritical carbon dioxide extraction [135], ultrasound-assisted extraction [136], and traditional solvent extraction followed by chromatographic methods [137,138]. Different extraction methods for fucoxanthin have been compared, and the extracted amount was variable [139]. In addition, other parameters such as solvent types, solvent volume, temperature, etc., are crucial in determining the concentration and purity of fucoxanthin. Therefore, optimum conditions of these parameters are necessary for obtaining the highest wield possible [140]. Several review articles have discussed in depth the extraction and purification of fucoxanthin. For example, carotenoids (i.e., fucoxanthin) extracted from algae using different innovative methods were summarized [141]. In addition, Lourenco-Lopes et al. (2020) discussed the available extraction, quantification, and purification methods with the purpose of recovering the highest ratio of fucoxanthin [142]. 

#### 6.1.4. Environment

Specific pigment fractions have been utilized to identify the algal types in the phytoplankton community. Fucoxanthin is a specific pigment in Bacillariophyceae and Chrysophyceae, whereas Pyrrophyta consists of peridinin as their unique pigment. Chlorophyll *b* and/or lutein are exclusive to Chlorophyta and Euglenophyta, while the specific pigment of Cyanophyta is myxoxanthophyll [143,144]. Pigment analysis using chromatographic methods provided reliable results of different major algal types in the phytoplankton community than microscopy enumeration [143]. Therefore, pigment analysis has been applied in the environmental study of algae. For example, Barlow (1995) [145] investigated the phytoplankton community in the western Alboran Sea based on pigment biomarkers. Recently, Wang et al. (2020) [146] reported the seasonal differences of the phytoneuston community structure in Daya Bay based on algal pigment analysis. The unique pigment of algae could also be employed to monitor the phytoplankton changes during the bloom of phytoplankton and determine the causative species for the bloom [147]. Moreover, the freshwater phytoplankton dynamics affected by seasonally variable freshwater inputs were investigated using pigment biomarkers [148]. Another study employed phytoplankton pigment profiling as a potential bioindicator of stratification conditions in lakes [149]. Apart from these, the significance of algal pigments was highlighted for paleolimnological research [150,151]. On the other hand, the algal pigment was also utilized to study the diet of zooplankton [152] and the impact of grazing by zooplankton on phytoplankton in the environment [153].

## 7. Commercial Products and Potential Applications of Fucoxanthin

**Figure 6:**
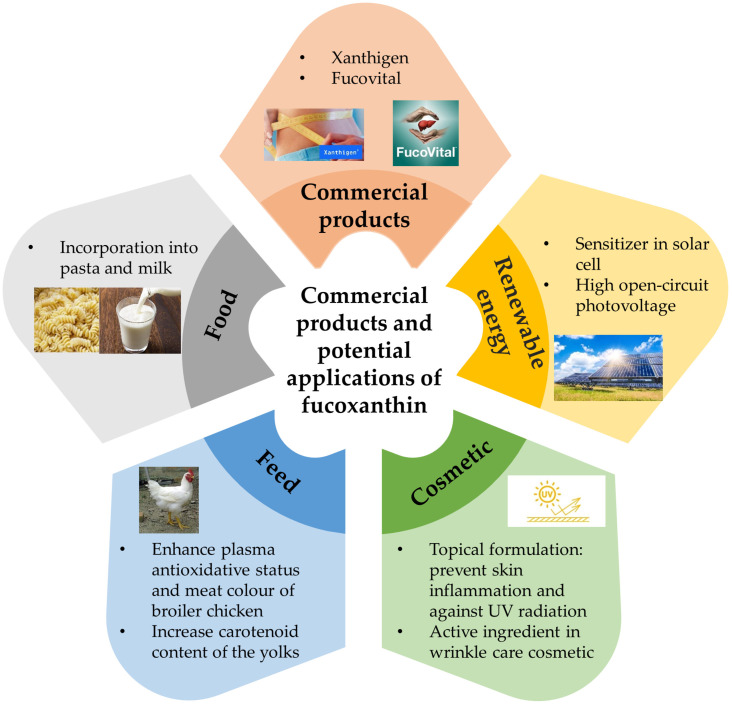
Commercial products and potential applications of fucoxanthin.

Until now, only two algae extracts containing fucoxanthin as commercial products are available in the market (Figure 6). Xanthigen is an antiobesity commercial product, which combined 0.4% fucoxanthin from brown macroalgae *Undaria pinnativida* and 35% punicic acid from pomegranate seed oil (https://nektium.com/branded-ingredient/xanthigen/, accessed on 30 July 2021). The antiobesity activity of this product was examined in a clinical trial [154]. The findings indicated the reduction of body and liver fat content and improved liver function in the subjects after orally administered Xanthigen for 16 weeks. The suppression of adipocyte differentiation and lipid accumulation by Xanthigen was via multiple mechanisms [155]. In addition, Xanthigen was demonstrated to be safe for consumption [156]. The other commercial product is FucoVital (https://www.algatech.com/algatech-product/fucovital/, accessed on 30 July 2021), which consists of 3% fucoxanthin, omega-3s, and other beneficial fatty acids (extracted from *P. tricornutum*). FucoVital is the first fucoxanthin food ingredient product to improve liver health that was approved by the United States Food and Drug Administration (NDI 1048, 2017).

Several studies demonstrated the potential in commercializing fucoxanthin. For renewable energy, brown macroalgae (*Sargassum wightii*) extract (contained fucoxanthin) was shown to be suitable as a sensitizer in a solar cell, as it is a low-cost and environmentally friendly alternative to the ruthenium metal complexes [157]. In addition, high open-circuit photovoltage was observed in the bio-photovoltaic devices fabricated with FCP complexes and titanium dioxide nanostructures [158]. These findings indicated the possibility of fucoxanthin to be exploited in solar cells at the commercial level.

On the other hand, the cosmetic properties of fucoxanthin have been illustrated in several studies. For instance, a topical formulation containing fucoxanthin has been developed that could prevent exacerbations related to skin inflammatory pathologies and protect skin against UV radiation [159]. In addition, solid lipid nanoparticle formulation loaded with fucoxanthin demonstrated the UV-blocking potential [160]. Furthermore, Kang et al. (2020) reported that fucoxanthin concentrate extracted from *P. tricornutum* could be utilized as an active ingredient in wrinkle care cosmetics [161].

The benefits of fucoxanthin as a feed to broiler chicken were highlighted in several studies. Sasaki et al. (2010) [162] demonstrated feeding fucoxanthin to the broiler chicken enhanced both the plasma antioxidative status and meat color of the chicken. Moreover, fucoxanthin also decreased the number of harmful microorganisms and regulated the antioxidant metabolism of the chicken meat [163]. The addition of 10–15% of brown macroalgae into the basal diet of laying hens augmented the carotenoid content of the yolks by 7.5–10-fold [164]. Apart from these, the incorporation of edible brown macroalgae (rich in fucoxanthin) into the semolina (wheat)-based pasta enhanced the nutritional value of the pasta [165]. Furthermore, Mok et al. (2016) developed whole milk and skimmed milk fortified with fucoxanthin [166]. These fucoxanthin incorporated products showed superior plasma absorption and organ tissue accumulation rates for fucoxanthin [167]. Overall, these studies revealed the possibility of fucoxanthin to be widely exploited at the commercial level in addition to the health supplements (Xanthigen and FucoVital).

## 8. Challenges and Possible Solutions

Recently, a broad spectrum of health benefits of fucoxanthin has been well illustrated. Thus, the demand for fucoxanthin is increasing rapidly. To fulfill the demand, the commercial production of fucoxanthin is a necessity. However, there are several challenges in fucoxanthin production. The amount of fucoxanthin produced using chemical synthesis is insufficient to meet the demand of the fucoxanthin market [168]. Hence, the fucoxanthin source has been shifted to natural sources such as brown macroalgae [40] and microalgae [111]. Microalgae are preferred as fucoxanthin sources due to their superior fucoxanthin content [111]. Commercial viability in fucoxanthin production was only examined in a few microalgae strains [169]. A comprehensive screening of high fucoxanthin-producing microalgae should be adopted globally via international collaboration. A further selection is needed to ensure the consistent production of biomass and fucoxanthin under variable conditions when cultivated in either the laboratory or outdoors [170]. Commercial fucoxanthin production relies on cultivation, extraction, and purification parameters. The optimization of these parameters is needed to enhance fucoxanthin production. With the optimized parameters, a standardized fucoxanthin production protocol could be developed to achieve a cost-effective production of fucoxanthin. Although biosynthesis pathways of fucoxanthin have been reviewed extensively [127,128,129], some pathways remain obscure due to the lack of experimental validation of these enzymes, intermediate products, and pathways [171]. Utilizing “omics” techniques could offer detailed insight into the ambiguous metabolic pathways and regulatory mechanisms in producing fucoxanthin [172].

Quality control is important in ensuring the safety, efficacy, and quality of a particular product. Heavy metal, pesticides, and other chemical contaminations might be present in the microalgae samples. Thus, monitoring the level of these contaminations according to the World Health Organization and the United States Food and Drug Administration is essential. The poor stability, low solubility, and weak bioaccessibility of fucoxanthin pose a challenge when incorporating fucoxanthin into food or supplements. The usage of nano/micro-encapsulation of fucoxanthin improved its stability and bioavailability [173,174]. Furthermore, most of the studies examined fucoxanthin effects only for a short duration [154]. Some bioactive compounds, such as alginates, have short period effects [175]. The effect of fucoxanthin might be overestimated regarding long-term effects. Thus, more intensive and longer studies, particularly human trials, are required to develop and verify fucoxanthin’s “true” effects.

## 9. Conclusions

The research trends of fucoxanthin from 1928 to June 2021 were analyzed in the present study. A significant increase in fucoxanthin publications, especially in recent years, was observed. This trend corroborates with the increased market demand for fucoxanthin over recent years (Global Fucoxanthin Market 2020). According to the bibliometric analysis, the three most productive countries were Japan (379 articles), the United States (319 articles), and China (230 articles). The top productive institutions were led by Hokkaido University, Japan (110 articles), followed by the CNRS Centre National de la Recherche Scientifique, France (61 articles), and Goethe-Universität Frankfurt am Main, Germany (49 articles). The analysis revealed that the United States and Germany were significantly intertwined within the global research network. Miyashita, Kazuo from Japan was the most productive and impactful author on fucoxanthin research with 88 publications and a Scopus *h*-index of 50. Most of the fucoxanthin articles were published in the *Marine Drugs* journal (123 articles) and the agricultural and biological sciences category (27.0%). Based on the keyword analysis, the research concepts of fucoxanthin could be divided into four clusters: bioactivities of fucoxanthin, photosynthesis, optimization of process, and environment. The bioactivities of fucoxanthin were identified as the potential future direction as the number of these studies increased tremendously over the years. The current study revealed the research trends of fucoxanthin and offered valuable information (i.e., most productive author, institutions, country) to establish effective global collaborations in studying fucoxanthin. In addition, the current findings could be utilized as fundamental in constructing impactful fucoxanthin research to expand the scientific knowledge on this unique carotenoid.

## Figures and Tables

**Table 1:** Top ten productive countries in producing fucoxanthin publications from 1928 to June 2021.

Country	TP	Number of Publications					
		1928–1970	1971–1980	1981–1990	1991–2000	2001–2010	2011–2021 *
Japan	379	0	4	13	37	98	227
United States	319	8	16	31	71	78	115
China	230	0	0	1	1	9	219
Germany	181	0	1	3	32	45	100
France	144	1	0	4	32	48	59
United Kingdom	132	5	6	5	28	26	62
South Korea	127	0	0	0	1	16	110
Spain	99	0	0	1	9	22	67
India	98	0	0	0	3	11	84
Canada	75	0	2	5	19	25	24

TP: Total publications; 2021 *: up to June 2021.

**Table 2:** Top ten productive institutions in producing fucoxanthin publications from 1928 to June 2021.

Institution	Country	TP	Number of Publications					
			1928–1970	1971–1980	1981–1990	1991–2000	2001–2010	2011–2021 *
Hokkaido University	Japan	110	0	0	1	8	33	68
CNRS Centre National de la Recherche Scientifique	France	61	1	0	1	17	17	25
Goethe-Universität Frankfurt am Main	Germany	49	0	0	0	3	13	33
Norges teknisk-naturvitenskapelige universitet	Norway	44	0	7	12	12	8	5
Chinese Academy of Sciences	China	41	0	0	1	1	2	37
Sorbonne Universite	France	39	0	0	1	10	14	14
Kyoto University	Japan	34	0	0	3	8	9	14
Kobe University	Japan	34	0	0	0	1	7	26
Plymouth Marine Laboratory	United Kingdom	33	0	0	1	15	6	11
Pukyong National University	Korea	33	0	0	0	1	2	30

TP: Total publications; 2021 *: up to June 2021.

**Table 3:** Top ten prolific authors in producing fucoxanthin research from 1928 to June 2021.

Author	Country	TP	Scopus *h*-Index	Scopus ID	Number of Publications					
					1928–1970	1971–1980	1981–1990	1991–2000	2001–2010	2011–2021 *
Miyashita, Kazuo	Japan	88	50	55883552800	0	0	0	0	29	59
Hosokawa, Masashi	Japan	68	44	7202009871	0	0	0	1	25	42
Buchel, Claudia	Germany	41	34	7006466104	0	0	0	0	13	28
Maeda, Hayato	Japan	31	19	8396571500	0	0	0	0	11	20
Liaaen-Jensen, Synnove	Norway	29	40	35509195900	0	6	11	10	2	0
Nagao, Ryo	Japan	28	21	22941377100	0	0	0	0	2	26
Akimoto, Seiji	Japan	23	27	7102347852	0	0	0	0	0	23
Shen, Jian Ren	Japan	22	50	56374284200	0	0	0	0	0	22
Maoka, Takashi	Japan	19	37	7004037866	0	0	0	1	5	13
Hashimoto, Hideki	Japan	18	44	35253778600	0	0	0	0	2	16

TP: Total publications; 2021 *: up to June 2021.

**Table 4:** Top ten prolific subject categories of fucoxanthin research from 1928 to June 2021.

Subject Category	TP (%)
Agricultural and Biological Sciences	27.0
Biochemistry, Genetics and Molecular Biology	18.1
Pharmacology, Toxicology and Pharmaceutics	8.3
Chemistry	7.9
Environmental Science	7.8
Earth and Planetary Sciences	7.5
Medicine	5.8
Chemical Engineering	3.8
Immunology and Microbiology	2.5
Engineering	2.3

TP: Total publications.

**Table 5:** Top ten core journals for research on fucoxanthin from 1928 to June 2021.

Journal	Publisher	IF (2020)	TP	TP (%)	Cummulative TP (%)
*Marine Drugs*	MDPI	5.118	123	5.91	5.91
*Journal of Phycology*	Wiley Periodical LLC	2.923	67	3.22	9.13
*Journal of Applied Phycology*	Springer	3.215	55	2.64	11.77
*Photosynthesis Research*	Springer	3.573	39	1.88	13.65
*Biochimica Et Biophysica Acta (BBA)—Bioenergetics*	Elsevier B.V.	3.991	38	1.83	15.48
*Algal Research*	Elsevier B.V.	4.401	32	1.54	17.02
*Marine Ecology Progress Series*	Inter-Research Science	2.824	30	1.44	18.46
*Journal of Agricultural and Food Chemistry*	American Chemical Society (ACS)	5.279	30	1.44	19.90
*Journal of Plankton Research*	Oxford University Press (OUP)	2.455	24	1.15	21.05
*Marine Biology*	Springer	2.573	24	1.15	22.20

MDPI: Multidisciplinary Digital Publishing Institute; IF: Impact factor; TP: Total publications.

**Table 6:** Top ten most cited fucoxanthin publications from 1928 to June 2021.

Title	Authors	Year	Journal	Total Citation
Fucoxanthin from edible seaweed, *Undaria pinnatifida*, shows antiobesity effect through UCP1 expression in white adipose tissues	Maeda et al.	2005	*Biochemical and Biophysical Research Communications*	411
Fucoxanthin, a marine carotenoid present in brown seaweeds and diatoms: Metabolism and bioactivities relevant to human health	Peng et al.	2011	*Marine Drugs*	356
Fucoxanthin as the major antioxidant in *Hijikia fusiformis*, a common edible seaweed	Yan et al.	1999	*Bioscience, Biotechnology and Biochemistry*	348
Radical scavenging and singlet oxygen quenching activity of marine carotenoid fucoxanthin and its metabolites	Sachindra et al.	2007	*Journal of Agricultural and Food Chemistry*	308
Fucoxanthin induces apoptosis and enhances the antiproliferative effect of the PPARγ ligand, troglitazone, on colon cancer cells	Hosokawa et al.	2004	*Biochimica et Biophysica Acta (BBA)—General Subjects*	261
Dietary combination of fucoxanthin and fish oil attenuates the weight gain of white adipose tissue and decreases blood glucose in obese/diabetic KK-A^y^ mice	Maeda et al.	2007	*Journal of Agricultural and Food Chemistry*	226
A potential commercial source of fucoxanthin extracted from the microalga *Phaeodactylum tricornutum*	Kim et al.	2012	*Applied Biochemistry and Biotechnology*	218
Evaluation of anti-inflammatory effect of fucoxanthin isolated from brown algae in lipopolysaccharide-stimulated RAW 264.7 macrophages	Heo et al.	2010	*Food and Chemical Toxicology*	212
Fucoxanthin inhibits the inflammatory response by suppressing the activation of NF-κB and MAPKs in lipopolysaccharide-induced RAW 264.7 macrophages	Kim et al.	2010	*European Journal of Pharmacology*	204
Production, characterization, and antioxidant activity of fucoxanthin from the marine diatom *Odontella aurita*	Xia et al.	2013	*Marine Drugs*	178

## Data Availability

Not applicable.
